# The relationship between health literacy and empowerment in pregnant women: a cross-sectional study

**DOI:** 10.1186/s12884-022-04686-z

**Published:** 2022-04-22

**Authors:** Nikta Tavananezhad, Amjad Mohamadi Bolbanabad, Fatemeh Ghelichkhani, Fatemeh Effati-Daryani, Mojgan Mirghafourvand

**Affiliations:** 1 Department of Obstetrics, Islamic Azad University, Sanandaj Branch, Sanandaj, Iran; 2 Healthcare Services Management, Social Determinants of Health Research Center, Research Institute for Health Development, Kurdistan University of Medical Sciences, Sanandaj, Iran; 3 Department of Midwifery, Imam Sajjad Hospital, Shahriar, Iran University of Medical Sciences, Shahriar, Tehran Islamic Republic of Iran; 4 Midwifery Department, Faculty of Nursing and Midwifery, Reproductive Health Research Center, Urmia University of Medical Sciences, Urmia, Iran; 5 Social Determinants of Health Research Center, Faculty of Nursing and Midwifery, Tabriz University of Medical Sciences, Tabriz, Iran

**Keywords:** Health Literacy, Empowerment, Pregnant Women

## Abstract

**Background:**

Maternal health literacy is defined as the acquisition of required cognitive and social skills to enable women to access, understand, appraise, and use the information needed to maintain and enhance their health conditions. The World Health Organization (WHO) proposes health literacy and women empowerment as two pivotal components of maternal health improvement programs. In this regard, providing women with education and training in various fields is a key factor for their empowerment, prosperity, and well-being. Therefore, the present study aimed to determine the relationship between health literacy and empowerment during pregnancy.

**Methods:**

This descriptive-analytical cross-sectional study examined 355 pregnant women, presented to different health centers in Sanandaj, Iran, in 2021. The cluster technique was used for sampling. For data collection, the socio-demographic and obstetrics characteristics, health literacy, and pregnant women's empowerment questionnaires were completed by interviewing research subjects. Data analysis was done using t-test, one-way ANOVA, Pearson correlation coefficient, and multivariate linear regression in STATA13.

**Results:**

The mean and standard deviation of health literacy and empowerment were 80.03 ± 12.79 (0–100) and 80.30 ± 8.14 (27–108), respectively. In terms of empowerment, the highest (19.50) and the lowest (12.92) scores were, respectively, observed in subdomains of “self-efficacy” and “the joy of an addition to the family.” With respect to health literacy, the highest (88.52) and lowest (73.78) mean scores were, respectively, observed in the subdomains of “understanding” and “access.” Pearson correlation test suggested that there was a significant direct correlation between the overall health literacy (*r* = 0.26; *p* < 0.001) and access (*r* = 0.18; *p* = 0.001), understanding (*r* = 0.11; *p* = 0.038), evaluation (*r* = 0.18; *p* = 0.001), and decision-making (*r* = 0.33; *p* < 0.001) with empowerment during pregnancy. Based on the multivariate linear regression model, empowerment during pregnancy improved with increasing health literacy (B = 0.16, 95% CI = 0.09 to 0.23; *p* < 0.001).

**Conclusion:**

The results show a direct relationship between health literacy and its dimensions with empowerment during pregnancy. Therefore, it is recommended to improve the health literacy of all women of reproductive age.

## Background

Based on a definition by Sørensen et al., health literacy is correlated with education and entails people's knowledge, motivation, and competency to access, understand, appraise, and apply health information for making decisions in healthcare, disease prevention, and health promotion with the ultimate goal of maintaining or improving their quality of life [1]. With the recent improvements in health status and health care, people have new health-related needs that require them to make decisions to meet those needs [2]. In an ideal situation, health literacy can enable people to acquire the necessary health information from valid and reliable sources and cooperate with health care providers and the community to improve their health status [3]. In particular, maternal health literacy can be defined as obtaining cognitive and social skills, which empower women to better access, evaluation, understanding, and use of the information to improve and maintain their health, and get prepared for childbirth and parenting [4, 5].

Recently, many studies have been conducted on health literacy and its impact on pregnancy and childbirth [6–9]. As an example, a review study (2021) showed that high levels of health literacy in pregnant women have a positive effect on their anxiety level and tobacco use, proper use of prescribed drugs, and lifestyle during pregnancy. This study revealed that in contrast to the level of health literacy of pregnant women in high-income western countries, health literacy is limited in countries below the poverty line [8]. Asadi et al. (2020) investigated pregnant women presented to health centers in Yazd, Iran, and found that they had a low level of health literacy [6]. In contrast, Endres et al. found that only 22% of pregnant women had low levels of health literacy [7].

Given the escalating emphasis on the improvement of women’s health, the main focus of international efforts is to achieve equitable health care through women empowerment [10, 11]. Moreover, women's empowerment is essential for the comprehensive development of society. Therefore, "women's empowerment" has become one of the core programs and activities of governmental and non-governmental organizations in recent years [12]. WHO proposes education and promotion of health literacy and empowerment of women as pivotal components of maternal health improvement programs to achieve a sustainable future, encourage further research, reform policies and laws, and raise awareness in this context [13]. In this regard, the results suggested education and training in various fields as the key factors for women's empowerment, prosperity, and well-being. Therefore, increasing women's educational attainment is very important in this regard [14].

However, defining and understanding the concept of empowerment continue to remain complicated [15]. Gibson (1991) explained the concept of midwife and nurse empowerment as follows: The process of recognizing and enhancing women's ability to meet health needs, solve health problems, and take action to feel in control of their health and lives [16]. Kameda’s defined empowerment during pregnancy as a sense of self-actualization and increasing independence through interacting with the environment and other people, which ultimately leads to a spontaneous increase in psychological energy and desirable pregnancy and childbirth [17].

The literature results indicate that maternal empowerment leads to increased use of contraceptive methods, receiving prenatal care, reduced rate of unsafe abortion, improved quality of newborn care, reduced rate of child mortality, complete vaccination, and generally improved pregnancy and childbirth health [11, 18, 19]. Despite the importance of women's empowerment, there are scant studies on the degree of pregnant women's empowerment in different parts of the world. Among these studies, a study conducted in Vietnam reported an optimal degree of empowerment in pregnant women [20]. The result of this study was consistent with some studies conducted in Iran [21–24].

Thus, regarding the particular importance of the subject, the inconsistencies in the literature results, and the significance of two components of health literacy and empowerment in promoting maternal health, this study aimed at investigating the relationship between health literacy and pregnant women empowerment in Sanandaj, Iran.

## Methods

### Study design and participants

This cross-sectional study was conducted on 355 pregnant women presented to health centers in Sanandaj, Iran, in 2020, to investigate the relationship between health literacy and empowerment of pregnant women. The inclusion criteria were pregnancy and the inclination to participate in the study. The exclusion criteria were an uncompleted questionnaire (at least 20%) and having a mental disability to respond to the questions.

Based on the findings of Ghanbari et al. [25], the calculated sample size was 237 according to the largest standard deviation related to the subdomain of reading comprehension of health literacy (SD = 12.42), with a precision (d) of 0.05 around the mean (m = 34.44), and α = 0.05. The final sample size was 355 based on the cluster sampling and considering the design effect of 1.5.

### Sampling

The cluster technique was used for sampling. First, half of the health centers in Sanandaj were randomly selected, and a list of potentially eligible women was extracted from the integrated health system (SIB). The number of the selected pregnant women in each center was proportional to the number of eligible women in that center. The subjects were randomly selected from the list at www.random.org. The subjects were contacted via phone, briefly explained the research objectives and methodology, and asked to attend the health center at a certain time if they intended to participate. The study objectives were fully explained in a face-to-face meeting and data collection instruments were completed.

### Instruments

The data collection instruments were the socio-demographic and obstetrics characteristics, health literacy, and empowerment questionnaires.

**The socio-demographic checklist** included items on age, body mass index (BMI), gravidity, history of infertility, educational attainment, employment status, economic status, and medical history.

**The health literacy questionnaire** was “Health Literacy for Iranian Adults.” This questionnaire was developed and psychometrically examined by Montazeri et al. (2014). Its content and face validities were assessed qualitatively. The construct validity was assessed using the exploratory factor analysis and the Kaiser–Meyer–Olkin (KMO) value was 0.919 at a significant level of *p* < 0.001. The explained variance for the 5-factor structure was about 53.2%. Cronbach’s alpha was used for measuring the reliability of the total scale and its subdomains, which were between 0.72 to 0.89 [26]. This questionnaire’s items included five domains of reading skills with 4 questions (i.e. “Reading educational materials about health (booklets, pamphlets, leaflets) is easy for me”), accessing with 6 questions (i.e. “I can find health information from different sources when I need such information”), understanding with 7 questions (i.e. “I can understand signage guidelines in hospitals, clinics, and health centers”), evaluation with 4 questions (i.e. “I can evaluate health-related information on the Internet”), and decision making and behavior with 12 questions (i.e. “I use a seat belt when driving, I visit my physician for regular checkups”) [26]. All the items were scored on a Likert scale, anchored by 1 to 5 (1 = never, 2 = rarely, 3 = sometimes, 4 = usually, 5 = always). The score of each domain is calculated separately, and an overall score is obtained for all domains. Health literacy in this study was categorized at four levels, namely “inadequate (0–50),” “not adequately sufficient (50.1–66),” “sufficient (66.1–84),” and “excellent (84.1–100).” The raw score of each domain is obtained from the sum of the scores of each domain’s items. To convert this raw score into the range of 0 to 100, the "minimum possible raw score" in each domain was subtracted from the raw score and then divided it into the subtraction of the minimum and maximum possible raw score in that domain. To obtain the overall health literacy score, the scores of all domains (in the range of 0–100) were summed up and divided by the number of domains (5).

The **pregnant women's empowerment questionnaire** was developed by Kameda et al. (2008) [17]. It is comprised of 27 items in five domains of self-efficacy with six questions (i.e. “I can probably deal with what I am worried about”), future image with six items (i.e.. “I have my ideal image of childbirth”), self-esteem with seven items (i.e. “I probably can deliver like other people”), support and assurance from others with four items (i.e. “I can ask help when I need to”), and joy of an addition to the family with four items (i.e. “I am looking forward to life after childbirth”). This questionnaire is scored on a four-point Likert scale, ranging from 1 (strongly disagree) to 4 (strongly agree). The minimum and maximum scores are 27 and 108, respectively. A high score indicates high pregnancy empowerment. The validity and reliability of the questionnaire were examined by Hajipour et al. in Iran. The validity and reliability of the questionnaire were approved using the content validity and Cronbach’s alpha (0.89), respectively [24].

### Data analysis

Descriptive statistics, including mean ± standard deviation and frequency (percentage), were used to assess socio-demographic characteristics, health literacy, and empowerment. The independent t-test (for binary scales) and one-way ANOVA (for ordinal and nominal scales) were used to assess health literacy and empowerment at different levels of the independent variables. The Pearson correlation was used to investigate the relationship between independent variables (with Interval and ratio scales), health literacy, and empowerment. Multivariate linear regression was applied to the variables correlated with empowerment with a *p*-value < 0.2, based on the univariate linear regression. The data was analyzed in STATA13 at a significance level of *p *< 0.05.

## Results

### Sociodemographic characteristics

Overall, 353 pregnant women with a mean ± SD age of 29.68 ± 5.86 years and a mean ± SD marriage length of 7.08 ± 5.02 years participated in this study. According to the findings, 60.3% of the participants (*n* = 213) had secondary and high school education, and only 16.1% of them (*n* = 57) were employed. The majority (61.1%) of them reported moderate economic status. According to Table 1, 5.4% of the participants (*n* = 19) had a history of infertility.

**Table 1:** Socio-demographic variables of pregnant women

Variables	N (%)^a^	Health literacy		Empowerment^‡^	
		**Mean (SD**^b^**)**	***P*-value**	**Mean (SD**^**b**^**)**	***P*-value**
**Total**	**353 (100)**	**80.03 (12.79)**	**-**	**80.30 (8.14)**	**-**
**Education**					
Illiteracy and elementary	40 (11.3)	79.68 (17.07)		78.36 (10.11)	
Middle and high school	213 (60.3)	79.71 (12.81)	0.772^* *^	81.38 (8.32)	0.012^* *^
Academic	100 (28.3)	80.81 (10.72)		78.80 (6.40)	
**Education of husband**					
Illiteracy and elementary	44 (12.5)	77.81 (18.39)		81.57 (10.24)	
Middle and high school	215 (60.9)	80.36 (12.31)	0.513^* *^	80.59 (8.28)	0.205^* *^
Academic	94 (26.6)	80.25 (10.84)		79.03 (6.56)	
**Employment**					
Employed	57 (16.1)	77.59 (16.19)	0.113^††^	78.98 (8.04)	0.202^††^
Housewife	296 (83.9)	80.53 (11.93)		80.56 (8.15)	
**Type of husband employment**					
Worker	93 (26.3)	78.43 (14.88)		81.83 (9.07)	
Employee	63 (17.8)	81.15 (9.49)	0.357^* *^	79.10 (5.88)	0.106^* *^
Others^c^	197 (55.8)	80.43 (12.76)		80.00 (8.27)	
**Status of family economic**					
Moderate	215 (61.1)	80.32 (11.83)		80.19 (7.66)	
High	46 (13.1)	78.21 (16.87)	0.564^* *^	78.44 (6.91)	0.111^* *^
Low	91 (25.9)	80.54 (12.44)		81.59 (9.59)	
**History of illness in pregnancy**					
Yes	16 (4.5)	79.14 (12.06)	0.556^††^	79.12 (7.50)	0.242^††^
No	337 (95.5)	80.22 (12.92)		80.53 (8.26)	
**Number of Pregnancy**					
1	162 (45.9)	79.52 (13.81)		80.14 (7.73)	
2	132 (37.4)	79.05 (12.54)	0.059^* *^	80.33 (8.19)	0.920^* *^
≥ 3	59 (16.7)	83.74 (9.15)		80.66 (9.22)	
**History of infertility**					
Yes	19 (5.4)	74.15 (9.72)	0.044^††^	75.44 (5.36)	0.009^††^
No	334 (94.6)	80.36 (12.85)		80.58 (8.19)	
	**Mean (SD**^**b**^**)**	**r**	***P*-value**^**‡‡**^	**r**	***P*-value**^**‡‡**^
**Age**	29.68 (5.86)	-0.032	0.558	-0.190	0.001
**Duration of marriage**	7.08 (5.02)	0.031	0.576	-0.21	0.001
**Body mass index**	27.94 (3.79)	-0.019	0.728	-0.032	0.560

^a^N (%): Number (percent); ^b^*SD* Standard Deviation; ^c^Others refer to Freelancer jobs; Score range of health literacy is between 0 to 100; ^‡^ Score range of empowerment is between 27 to 108; ^* *^One-way ANOVA; ^††^ Independent t-test; ^‡‡^ Pearson correlation test

### Health literacy

The mean ± SD of the health literacy among the pregnant women in the study was 80.03 ± 12.79 (in the range of 0–100). Among the health literacy domains, the highest and the lowest mean ± SD were for the subdomains of “understanding” (88.52 ± 13.48) and “access” (73.78 ± 17.52), respectively (Table 2).

**Table 2:** Mean score of health literacy and empowerment with their dimensions in pregnant women

**Variables**	**Minimum**	**Maximum**	**Mean**	**SD**^a^	**Correlation with empowerment**	
					r	*P*-value^†^
Reading	0.0	100.0	82.12	17.60	0.03	0.543
Access	0.0	100.0	73.78	17.52	0.18	**0.001**
Understand	10.71	100.0	88.52	13.48	0.11	**0.038**
Evaluation	18.75	100.0	78.76	16.97	0.18	**0.001**
Decision-making	25.0	100.0	77.54	15.65	0.33	** < 0.001**
**Health literacy** ^b^	13.63	100.0	80.03	12.79	0.26	** < 0.001**
Self-efficacy	6.0	24.0	18.35	2.86	-	-
Picture of future	7.0	24.0	16.15	2.78	-	-
Self-esteem	14.0	25.0	19.50	2.08	-	-
Support and approval	8.0	16.0	13.33	1.89	-	-
The pleasure of adding someone to the family	8.0	16.0	12.92	1.73	-	-
**Empowerment**^**c**^	58.0	104.0	80.30	8.14	-	-

^†^Pearson correlation test; ^a^*SD* Standard Deviation; ^b^Score range of health literacy and their dimensions are between 0 to 100; ^c^Score range of empowerment is between 28 to 108 and score of their dimensions are self-efficacy (6 to 24), picture of future (6 to 24), self-esteem (7 to 28), support and approval from others (4 to 16), pleasure of adding someone to the family (4 to 16)

### Pregnant women’s empowerment

The mean ± SD of the participants’ empowerment during pregnancy was 80.30 ± 8.14 (in the range of 27–108). According to Table 2, the highest and lowest scores were for subdomains of “self-efficacy” (19.50) and “the joy of an addition to the family” (12.92), respectively.

### Relationship between health literacy and empowerment during pregnancy

Pearson correlation test (Table 2) showed a significant direct relationship between the health literacy score ( *r* = 0.26; *p* < 0.001), and the domains of access (*r* = 0.18; *p* = 0.001), understanding (*r* = 0.11; *p* = 0.038), evaluation (*r* = 0.18; *p* = 0.001), and decision-making (*r* = 0.33; *p* < 0.001) with empowerment during pregnancy.

Table 3 shows the variables, correlated with empowerment with a *p* < 0.2 in the univariate linear regression, such as health literacy, age, educational attainment, employment status, spouse employment status, family economic status, history of illness in pregnancy, and history of infertility. These variables were inputted into the multiple linear regression model and the model with the highest R^2^ was determined (R^2^ = 0.153). Among the variables inputted into the model, health literacy (coefficient = 0.17, 95% CI: 0.10 to 0.24; *p* < 0.001) was positively correlated with empowerment during pregnancy. In contrast, age (coefficient = -0.20, 95% CI: -0.35 to -0.05) was inversely correlated with empowerment (Table 4).

**Table 3:** Univariate liner regression model for predicting pregnancy empowerment of pregnant women

***Variable***	***B (coefficient)***	***Std.B***	***T***	***P-value***	***95% CI***^a^
**Health literacy**	0.17	0.03	4.87	< 0.001	0.10, 0.24
**Age**	-0.26	0.07	-3.50	0.001	-0.41, -0.11
**Body mass index**	-0.07	0.12	-0.58	0.560	-0.31, 0.16
**Education**					
Illiteracy and elementary	*Ref*^b^				
Middle and high school	3.07	1.42	2.11	0.035	0.21, 5.82
Academic	0.44	1.55	0.28	0.777	-2.60, 3.48
**Education of husband**					
Illiteracy and elementary	*Ref*^b^				
Middle and high school	-0.98	1.43	-0.69	0.494	-3.81, 1.84
Academic	-2.54	1.58	-1.61	0.201	-5.65, 0.57
**Employment**					
Employed	-1.57	1.21	-1.30	0.193	-3.96, 0.80
Housewife	*Ref*				
**Type of husband employment**					
Worker	*Ref*^b^				
Employee	-2.73	1.37	-1.99	0.048	-5.43, -0.02
Others^+^	-1.82	1.07	-1.70	0.089	-3.93, 0.28
**Status of family economic**					
Low	*Ref*^b^				
Moderate	-1.75	1.36	-1.29	0.198	-4.44, 0.92
High	1.39	1.05	1.32	0.186	-0.68, 3.47
**History of illness in pregnancy**					
Yes	*Ref*^b^				
No	-3.43	2.08	-1.65	0.101	-7.52, 0.66
**Number of Pregnancy**					
1	*Ref*^b^				
2	0.18	0.99	0.19	0.849	-1.76, 2.14
≥ 3	0.51	1.27	0.41	0.686	-1.99, 3.02
**History of infertility**					
Yes	*Ref*^b^				
No	5.13	1.95	2.62	0.009	1.28, 8.98

^a^*CI* Confidence interval; ^b^Reference

**Table 4:** Multivariate liner regression model for predicting pregnancy empowerment of pregnant women

*Variable*	*B (coefficient)*	*Std.B*	*T*	*P-value*	*95% CI*^*a*^
**Constant**	72.55	4.84	14.98	< 0.001	63.02, 82.08
**Health literacy**	0.17	0.03	4.87	** < 0.001**	0.10, 0.24
**Age**	-0.20	0.75	-2.67	**0.008**	-0.35, -0.05
**Education**					
Illiteracy and elementary	*Ref*^b^				
Middle and high school	2.47	1.45	1.70	0.091	-0.39, 5.33
Academic	0.59	1.72	0.34	0.731	-2.80, 3.99
**Employment**					
Employed	0.03	1.23	0.03	0.980	-2.40, 2.47
Housewife	*Ref*^b^				
**Type of husband employment**					
Worker	*Ref*^b^				
Employee	-1.78	1.62	-1.10	0.272	-4.97, 1.40
Others^+^	-2.24	1.11	-2.00	**0.046**	-4.44, -0.04
**Status of family economic**					
Low	*Ref*^b^				
Moderate	-0.67	1.34	-1.10	0.272	-4.97, 1.40
High	1.05	1.03	1.02	0.311	-0.98, 3.07
**History of illness in pregnancy**					
Yes	*Ref*^b^				
No	-2.70	1.97	-1.37	0.172	-6.59, 1.18
**History of infertility**					
Yes	*Ref*^b^				
No	2.96	1.95	1.52	0.130	-0.87, 6.80

^a^*CI* Confidence interval; ^b^Reference; *R*^*2*^ = 0.153, *F* = 5.01, *p* < 0.001

## Discussion

The findings showed that pregnant women had high levels of health literacy. A significant positive relationship was observed between the overall health literacy score and its domains with empowerment during pregnancy. Based on the multiple linear regression model, a significant correlation was shown between health literacy and age with empowerment during pregnancy after adjusting the socio-demographic characteristics. In other words, empowerment during pregnancy improved with increasing health literacy and decreased with aging.

The results revealed a positive and significant relationship between health literacy and empowerment during pregnancy, which corroborates Bastani et al.’s findings [27] that education is directly correlated with learning and skills. Although the majority of the participants had less than a diploma, they had sufficient health literacy indicative of the awareness level of pregnant mothers. These findings indicate that health literacy is a positive factor in empowerment during pregnancy and education can enable women and help them develop their inner power [27, 28]. In fact, health literacy empowers individuals to play an active role in changing environments to affect health [29]. A review study (2016) revealed that women’s empowerment is significantly correlated with improvement in pregnancy and childbirth outcomes, better and planned pregnancy care by the mothers, and better nutritional state of the mothers [18]. In fact, these factors, namely better access and empowerment, help individuals get access, understand, and use the available information for their health improvement [3].

The results of the present study proposed age as an influential factor in pregnant women’s health literacy level. In other words, pregnant women's empowerment weakens with aging, which is inconsistent with the findings of the study by Ahmed et al. [30], who investigated the results of 33 developing countries, and also Batool et al.’s study conducted in Pakistan [31]. This inconsistency may be due to differences in the research contexts and the various factors, which affect pregnant women's empowerment in different societies.

Given the mean score of pregnant women empowerment, the results are consistent with the findings of Hajipour et al. [24] and Borghei et al. [22] in Iran. Among the empowerment domains, the highest score was for “self-esteem” and the lowest score was for “the joy of an addition to the family.” Moreover, the lowest score was for “support from others”, second to the "the joy of an addition to the family", indicating insufficient interaction and support of people around the pregnant mothers. The low score in “the joy of an addition to the family” may be indicative of an unwillingness to childbearing due to negative experiences of pregnancy and previous childbirth, economic problems, or even mothers with high educational attainment. Nilsson et al. [32] showed that family support during pregnancy can lead to positive experiences of mothers which, in turn, enhances mothers’ empowerment and self-esteem. A study conducted in Africa indicated that the extent to which women make family decisions independently has an effect on such factors as the number of children [33].

Maternal health literacy is a cognitive and social skill, which shows their motivation and ability to give adequate access, understanding, and use of information to keep themselves and their children healthy [34]. It is indicative of skills and resources, which demonstrate the ability of people in health information processing [35]. The results showed that pregnant women have sufficient levels of health literacy, which is consistent with the findings of Moshki et al. (2018) [36] and Ghanbari et al. (2020) [25] in Iran. In other words, to make appropriate decisions about health literacy, individuals need to understand and use the information provided to them in specific health centers. Therefore, the service providers should know the patients’ health information processing abilities to improve the outcome of their disease. Moreover, they should provide information to patients with different levels of health literacy [37]. The majority of health service providers in Iran share a similar cultural context and language with the pregnant women covered by them and have a close relationship with them, which can justify the sufficient health literacy of pregnant women living in Sanandaj.

In the present study, “understanding” and “accessing” obtained the highest and the lowest scores in health literacy domains, respectively. This is inconsistent with the findings of Izadirad et al. in Baluchestan-Iran (2019) [38], where 43 primigravida women were investigated and obtained the highest and the lowest health literacy scores in “decision-making” and “reading,” respectively. The possible explanation for this inconsistency is the differences in the study population. The majority of the participants in Izadirad’s study had less than secondary school education and low family income; whereas, most of the mothers in the present study had secondary and high school education and moderate economic status. By creating a culture and paying attention to health literacy in school, girls can be acquainted with health literacy and its outcomes from childhood. As a result, women with more understanding of health literacy gain more access to the services they need which, in turn, leads to their empowerment.

Among the strengths of the present study are the relatively large sample size and using random sampling, which increases the generalizability of the findings. The use of valid and reliable questionnaires for an Iranian population is another strength of the present study. The cross-sectional nature of this study is among its limitations because the discovered correlations do not precisely demonstrate a cause-and-effect relationship. Since various factors are involved with pregnant women’s empowerment, it is recommended to conduct more studies on this subject across different cultures. Another weakness is that it only included urban women living in urban areas of Sanandaj and thus the results may not be generalizable to rural women. It is then recommended to study the impact of various factors influencing the health literacy and empowerment of pregnant women in rural areas.

## Conclusion

The findings revealed a direct relationship between health literacy and its domains with empowerment during pregnancy. Regarding the particular significance of the Millennium Development Goals and growing global emphasis on promoting maternal and infant health, and also the significant impact of empowering pregnant women on the pregnancy and childbirth outcomes, maternal health literacy is an important and effective tool to increase the effectiveness of empowerment interventions and subsequently enhances the effectiveness of the care providers to achieve a healthy pregnancy outcome. Therefore, it is recommended to enhance the health literacy of all women at reproductive ages for further empowerment of pregnant women.

## Data Availability

The datasets generated and/or analysed during the current study are not publicly available due to limitations of ethical approval involving the patient data and anonymity but are available from the corresponding author on reasonable request.

## Abbreviations

SD: Standard Deviation
95% CI: 95% Confidence Interval
WHO: World Health Organization

## References

[1] Sørensen K, Van den Broucke S, Fullam J (2012). Health literacy and public health: a systematic review and integration of definitions and models. BMC Public Health.

[2] Nielsen-Bohlman L, Panzer AM, Kindig DA (2004). Health literacy: a prescription to end confusion.

[3] Chen X, Hay JL, Waters EA, Kiviniemi MT, Biddle C, Schofield E (2018). Health literacy and use and trust in health information. J Health Commun.

[4] Jordaan C (2009). A literature review on childbirth education: paediatrics. Prof Nurs Today.

[5] Renkert S, Nutbeam D (2001). Opportunities to improve maternal health literacy through antenatal education: an exploratory study. Health Promot Int.

[6] Asadi L, Amiri F, Safinejad H (2020). Investigating the effect of health literacy level on improving the quality of care during pregnancy in pregnant women covered by health centers. J Educ Health Promot.

[7] Endres LK, Sharp LK, Haney E, Dooley SL (2004). Health literacy and pregnancy preparedness in pregestational diabetes. Diabetes Care.

[8] Nawabi F, Krebs F, Vennedey V, Shukri A, Lorenz L, Stock S (2021). Health literacy in pregnant women: a systematic review. Int J Environ Res Public Health.

[9] Zibellini J, Muscat DM, Kizirian N, Gordon A (2021). Effect of health literacy interventions on pregnancy outcomes: a systematic review. Women Birth.

[10] Upadhyay UD, Gipson JD, Withers M, Lewis S, Ciaraldi EJ, Fraser A (2014). Women's empowerment and fertility: a review of the literature. Soc Sci Med.

[11] World Health Organization. New insights into quality of care for girls and women facing the complications of unsafe abortion. 2022; Available from: https://www.who.int/news/item/26-01-2022-new-insights-into-quality-of-care-for-girls-and-women-facing-the-complications-of-unsafe-abortion. Accessed in 8 Mar 2022.

[12] Mandal KC (2013). concept and types of women empowerment. Int Forum Teach Stud.

[13] World Health Organization. Standing up for sexual and reproductive health and human rights. 2020; Available from: https://www.who.int/news/item/19-11-2020-who-stands-up-the-right-to-health. Accessed in 8 Mar 2022.

[14] Sundaram MS, Sekar M, Subburaj A (2014). Women empowerment: role of education. Int J Manage Soc Sci.

[15] Hermansson E, Mårtensson L (2011). Empowerment in the midwifery context–a concept analysis. Midwifery.

[16] Gibson CH (1991). A concept analysis of empowerment. J Adv Nurs.

[17] Kameda Y, Shimada K (2008). Development of an empowerment scale for pregnant women. J Tsuruma Health Sci Soc Kanazawa Univ.

[18] Pratley P (2016). Associations between quantitative measures of women's empowerment and access to care and health status for mothers and their children: a systematic review of evidence from the developing world. Soc Sci Med.

[19] World Health Organization. Newborn Mortality. 2022; Available from: https://www.who.int/news-room/fact-sheets/detail/levels-and-trends-in-child-mortality-report-2021. Accessed in 8 Mar 2022.

[20] de Loenzien M, Mac QNH, Dumont A (2021). Women’s empowerment and elective cesarean section for a single pregnancy: a population-based and multivariate study in Vietnam. BMC Pregnancy Childbirth.

[21] Alishah A, Ganji J, Mohammadpour R, Kiani Z, Shahhosseini Z (2019). Women’s reproductive empowerment: a comparative study of urban and rural females in Iran. Int J Womens Health Reprod Sci.

[22] Borghei N, Taghipour A, Latifnejad Roudsari R, Jabbari NH (2017). Investigating the determinants of maternal empowerment during pregnancy: a strategy for prenatal healthcare promotion. J Midwifery Reprod Health.

[23] Borghei NS, Taghipour A, Roudsari RL, Keramat A, Noghabi HJ (2016). Predictors of prenatal empowerment among iranian pregnant women. Electron Physician.

[24] HajiPour L, Hosseini Tabaghdehi M, TaghiZoghi Z, Behzadi Z (2016). Empowerment of pregnant women. J Holist Nurs Midwifery.

[25] Ghanbari S, Majlessi F, Ghaffari M, Mahmoodi MM (2020). Evaluation of health literacy of pregnant women in urban health centers of Shahid Beheshti Medical University. Daneshvar Med.

[26] Montazeri A, Tavousi M, Rakhshani F, Azin SA, Jahangiri K, Ebadi M (2014). Health Literacy for Iranian Adults (HELIA): development and psychometric properties. Payesh.

[27] Bastani F, Haidarnia A, Vafaie M, Kazem-nejad A, Kashanian M (2006). The effect of relaxation training based on self-efficacy theory on mental health of pregnant women. Iran J Psychiatry Clin Psychol.

[28] Mullany BC, Hindin MJ, Becker S (2005). Can women's autonomy impede male involvement in pregnancy health in Katmandu, Nepal?. Soc Sci Med.

[29] Panahi R, Ebrahimi G, Ahmadi A (2018). Health literacy: a key component of controlling social determinants of health. J Educ Community Health.

[30] Ahmed S, Creanga AA, Gillespie DG, Tsui AO (2010). Economic status, education and empowerment: implications for maternal health service utilization in developing countries. PLoS One.

[31] Batool SS, Batool SA (2018). Levels of education, age, legal awareness and women's empowerment. J Res Reflect Edu.

[32] Nilsson L, Thorsell T, Hertfelt Wahn E, Ekström A (2013). Factors influencing positive birth experiences of first-time mothers. Nurs Res Pract.

[33] Upadhyay UD, Karasek D. Women's empowerment and achievement of desired fertility in sub-Saharan Africa; 2010. Avaiable at: https://ucghi.universityofcalifornia.edu/sites/default/files/pubs/whe-desired-fertility-in-sub-saharan-africa.pdf. Accessed in 23 Oct 2021.

[34] Mojoyinola J (2011). Influence of maternal health literacy on healthy pregnancy and pregnancy outcomes of women attending public hospitals in Ibadan, Oyo State. Nigeria African Res Rev.

[35] von Wagner C, Knight K, Steptoe A, Wardle J (2007). Functional health literacy and health-promoting behaviour in a national sample of British adults. J Epidemiol Community Health.

[36] Moshki M, Mirzania M, Kharazmi A (2018). The relationship of health literacy to quality of life and demographic factors in pregnant women: a cross-sectional study. J Health Literacy.

[37] Zaree F, Karimi F, Mohseni S, Mdani S, Dadipoor S, Mdani AH (2017). Health literacy of pregnant women and some related factors in pregnant women referred to Minab health centers. J Prevent Med.

[38] Izadirad H, Ali Ahmadi M, Niknami S (2019). Predicting factors influencing prenatal care based on health literacy in Balochistan primigravida women. J Health Literacy.

